# Doxycycline versus Curcumin for Inhibition of Matrix Metalloproteinase Expression and Activity Following Chemically Induced Inflammation in Corneal Cells

**DOI:** 10.18502/jovr.v19i3.13689

**Published:** 2024-09-16

**Authors:** Hamed Zareh, Alireza Shahriary, Ali Razei, Rouhollah Ameri, Mahdi Fasihi-Ramandi, Hossein Aghamollaei

**Affiliations:** ^1^Recombinant Proteins Department, Breast Cancer Research Center, Motamed Cancer Institute, ACECR, Tehran, Iran; ^2^Chemical Injuries Research Center, Systems Biology and Poisonings Institute, Baqiyatallah University of Medical Sciences, Tehran, Iran; ^3^Health Sciences and Technology Park, Mashhad University of Medical Sciences, Mashhad, Iran; ^4^Molecular Biology Research Center, Systems Biology and Poisonings Institute, Baqiyatallah University of Medical Science, Tehran, Iran; ^6^https://orcid.org/0000-0003-4275-8613; ^7^Hamed Zareh: https://orcid.org/0000-0002-6017-5270

**Keywords:** 2-Chloroethyl Ethyl Sulfide, Doxycycline, Curcumin, Inflammation, MMP-2, MMP-9

## Abstract

**Purpose:**

Sulfur mustard (SM) is a potent blistering agent. This alkylating chemical agent has extremely toxic effects on the eye. MMP-2 and MMP-9 are the two most important matrix metalloproteinase enzymes involved in the pathology of chemical eye injuries. Curcumin is regarded as a natural anti-inflammatory agent. This study aims to compare the anti-inflammatory effects of curcumin versus doxycycline on chemically induced corneal injuries.

**Methods:**

The HCE-2 cell line was used as a model for corneal cells. The effective concentrations of 2-chloroethyl ethyl sulfide (CEES) – as an analog of SM – doxycycline, and curcumin were determined using the MTT assay. The gene expression of *MMP-2*, *MMP-9*, and tissue inhibitors of metalloproteinase (TIMP-1) was evaluated by the real-time PCR method. Also, the activity of MMP-2 and MMP-9 enzymes was determined by zymography.

**Results:**

The expression of the MMP-2 and MMP-9 genes increased 5- and 3.3-fold after exposure to CEES, respectively. Following the treatment with curcumin and doxycycline, MMP-2 expression decreased significantly. Also, after treatment with curcumin and doxycycline, the MMP-9 expression decreased 2.5- and 1.6-fold, respectively. The reduction in activity was 32% for MMP-2 and 56% for MMP-9 after treatment with curcumin. The corresponding values were 12% and 40% following doxycycline treatment. There was no significant difference between the effects of curcumin and doxycycline on reducing MMP-2 expression, but the difference was statistically significant in the case of MMP-9.

**Conclusion:**

Doxycycline and curcumin can inhibit MMP expression and activity in chemically exposed corneal cells. Curcumin has a greater ability than doxycycline to inhibit MMP-2 and MMP-9 enzymes; however, the difference is statistically significant only in the case of MMP-9. After further validation, these substances can be introduced as anti- inflammatory agents to treat corneal chemical burns.

##  INTRODUCTION

Sulfur mustard (SM), or bis [2-chloroethyl] sulfide, is a potent and extremely reactive blistering agent.^[1]^ The use of SM as a chemical weapon in World War I and the Iran–Iraq War injured more than 210,000 people, 90% of whom developed chemical eye burns.^[2,3]^ SM is an alkylating chemical agent, and the eyes are the most susceptible organs to the toxic effects of this agent.^[3,4,5]^ Ocular damage occurs through various biochemical and molecular mechanisms. Studies have shown that the induction of severe inflammatory reactions in corneal layers plays an important role in the pathogenesis of SM.^[3,6,7]^

Another process that occurs during SM-induced eye damage is extracellular matrix (ECM) remodeling. ECM is mainly processed by matrix metalloproteinase (MMPs).^[6,8]^ MMPs are a family of zinc-dependent endopeptidases that are involved in the pathological processes in which ECM regeneration occurs and play a major role in tissue destruction.^[6,9]^ The two most important MMPs involved in the pathology of eye injuries are *MMP-2* and *MMP-9*.^[10,11,12]^ Since *MMP-2* (gelatinase A) and *MMP-9* (gelatinase B) are components of the basement membrane and denatured collagen (gelatin), they play a key role in the destruction of the basement membrane.^[13]^ ECM component degradation is controlled by the balance between MMPs and a group of tissue inhibitors of metalloproteinase (TIMPs).^[9,13]^ MMP inhibitors have been considered as a treatment for these injuries, and the family of tetracycline antibiotics such as doxycycline are effective inhibitors for MMPs.^[6]^ Doxycycline inhibits the activity of the MMPs through mechanisms such as inhibiting reactive oxygen species and restricting the expression of neutrophil collagenase and epithelial gelatinase genes.^[6,10,14,15]^

As the main phenolic and curcuminoid section of the *Curcuma longa*, curcumin is highly regarded as a natural anti-inflammatory agent.^[16]^ Curcumin's most important medicinal properties include its high antioxidants activity and anti-inflammatory properties.^[16]^ Because the FDA classifies curcumin as a GRAS substance (generally known as safe), it can be used to treat many ocular disorders such as dry eye syndrome and glaucoma.^[17]^ Considering the role of inflammation in chemical eye burns, curcumin may be an effective treatment for such injuries because of its impact on inflammatory reactions. This study aims to compare the effect of curcumin and doxycycline, as potential treatments for chemical eye burns, on the synthesis and activity of *MMP-2* and *MMP-9* in human corneal epithelial cells (HCE-2).

##  METHODS

### Cell Culture

The HCE-2 cell line was purchased from CELLnTEC Advanced Cell Systems AG (Switzerland). The culture medium consisted of keratinocyte serum-free medium (Gibco, UK), containing fetal bovine serum 10% (Sigma-Aldrich, Germany), insulin 0.005 mg/ml (Sigma-Aldrich, Germany), and penicillin 100 U/ml + streptomycin 100 µg/ml (Sigma-Aldrich, Germany). The cells were grown in a 10% CO ${\;}_{2}$ atmosphere at 37ºC.

The study protocol was reviewed and approved by the Ethics Committee at Baqiyatallah University of Medical Sciences, Tehran, Iran (No. IR.BMSU.REC.1398.072.).

### Cell Viability Assay and IC50 Calculation

The MTT assay was used to assess cell viability and IC50 calculations. In this method, 1 × 10^4^ cells in 100 µl of culture medium were seeded in a 96-well plate. After overnight incubation, the wells were divided into four groups: group 1 with 24 wells for doxycycline treatment (0.75–80 µg/ml), group 2 with 24 wells for curcumin treatment (0.75–80 µM), group 3 with 18 wells for CEES treatment (0.75–20 mM), and group 4 with 6 wells as the control group (only HCE-2 cells). Each concentration was evaluated three times. Forty-eight hours after cell treatment, 20 μ l of MTT (3-(4, 5-dimethylthiazol-2-yl)-2, 5-diphenyltetrazolium) was added and incubation was performed at 37ºC for 4 hours. Subsequently, the culture medium containing MTT was carefully removed, and 150 μ l of dimethyl sulfoxide (DMSO) was added to dissolve the formazan crystals.^[8]^ Finally, optical density was measured at 570 nm with a microplate reader, and cell viability percentage was calculated. The IC50 calculation was performed by GraphPad Prism.

### Determination of the Effective Dose of Doxycycline and Curcumin 

After the IC50 was calculated, the corneal cells (HCE-2 cells) were seeded in 96-well plates to determine the effective dose of doxycycline and curcumin. After overnight incubation, the cells were treated according to Table 1. The considered concentration for CEES was equivalent to IC50 (3.42 mM). Also, three concentrations lower than IC50 were considered for curcumin and doxycycline. Each concentration was evaluated three times. Forty-eight hours after cell treatment, 20 μ l of MTT was added and incubation was conducted at 37ºC for 4 hours. Subsequently, the culture medium containing MTT was carefully removed, and 150 μ l of DMSO was added to dissolve the formazan crystals. Finally, optical density was measured at 570 nm, and cell viability percentage was calculated.

### Preparation of Doxycycline Formulation

Having reviewed similar previous studies, we designed the formulation of the doxycycline product as a ready-to-dissolve powder at the time of use and a final product with 2% doxycycline. The ready-to-dissolve powder was considered to increase stability and maintain the maximum antioxidant power of the ophthalmic product [Table 2].

### Evaluation of MMP-2, MMP-9, and TIMP-1 gene expression

In this section, the expression of *MMP-2*, *MMP-9*, and *TIMP-1* genes in infected cells (by CEES) and treated cells (with doxycycline and curcumin) was measured using real-time PCR. For this purpose, 10^5^ HCE-2 cells were seeded in six-well plates. After overnight incubation, the cells were treated according to Table 3.

The concentrations of CEES, curcumin, and doxycycline were considered at 3.42 mM, 3 µM, and 10.11 µg/ml, respectively. Then, incubation was done for 24 hours at 37ºC and 5% CO ${\;}_{2}$ . Afterward, at first, total RNA was isolated from corneal epithelial cells through the acid-guanidinium thiocyanate-phenol-chloroform extraction method.^[15,18,19]^ Next, total RNA (1 µg) was reverse transcribed into cDNA using MuLV reverse transcriptase (50 U), oligo dT (50 pmol) as a primer, and RNAse inhibitor (20 U) at 42ºC for 30 minutes. For cDNA amplification, 2 µl of cDNA product, 0.4 µl of the relevant primers, 10 µl of SYBR Premix Ex Taq II (Takara, Japan), and 7.2 µl of DDW were mixed according to the manufacturer's protocol. The PCR reaction was initiated at 95ºC for 30 seconds, followed by 40 cycles of 95ºC for 10 seconds and 60ºC for 1 minute. These reactions were performed in the Applied Biosystem 7500 real-time quantitative PCR system (ABI, USA). The 18s rRNA gene was used as a housekeeping internal control. All real-time PCR reactions were performed in triplicate. The PCR primers for *MMP-2*, *MMP-9*, *TIMP-1*, and 18s rDNA are shown in Table 4. The relative expression of mRNA was calculated in comparison to 18s rRNA using the 2 ${\;}^{--\Delta \Delta Ct}$ method.^[20]^ The data are reported as the relative quantity of target mRNA compared to 18S rRNA mRNA.

### Analysis of MMP Activity (Zymography)

The activity of *MMP-2* and *MMP-9* in corneal cell culture was evaluated by gelatin zymography. For this purpose, the cultures were treated with CEES, doxycycline, and curcumin, the same way as in the sub-section entitled “Preparation of Doxycycline Formulation”. After 24 hours, the cultured cells were centrifuged and the supernatants were used for gelatin zymography assays. The supernatant of the corneal epithelial cultures with different treatments was electrophoresed on an 8% polyacrylamide gel containing 1% gelatin (Sigma-Aldrich, Germany). First, 5 to 20 ml of each medium was used, and the volume was adjusted so that the samples could have equal amounts (5 mg) of protein. The supernatants were mixed with SDS-PAGE sample buffer and run at 100 V for 90 minutes at 4ºC. The gel was then soaked with 0.25% Triton X-100 (SDS removal buffer) 30 minutes at room temperature and then incubated in enzyme activation buffer (50 mM Tris-HCl, 150 mM NaCl, 10 mM CaCl ${\;}_{2}$ , 0.02% Tween-20, and 5 mM PMSF) at 37ºC overnight to permit gelatinase (*MMP-2* and *MMP-9*) to digest its substrate. Subsequently, the gel was rinsed in distilled water, stained with Coomassie Brilliant Blue R-250, and then destained (7% v/v acetic acid, 5% v/v methanol). After photography, densitometric analysis of the bands (intensity and area) was conducted using the Image Station 4000MM (Kodak Digital Science, USA).

### Statistical Analysis

Data analysis was performed using the SPSS version 23 (IBM, USA), and the results were compared using the Student's *t*-test. The data are presented as mean ± SD, and a *P-value* < 0.05 was considered statistically significant.

**Table 1:** Treatment method to determine the effective dose of doxycycline and curcumin.


**Well no.**	**Treatment method and the contents of the wells**
1	10^4^ HCE-2 cells
2	10^4^ HCE-2 cells + 3.42 mM CEES
3	10^4^ HCE-2 cells + 3.42 mM CEES + 6.04 µM curcumin
4	10^4^ HCE-2 cells + 3.42 mM CEES + 3.02 µM curcumin
5	10^4^ HCE-2 cells + 3.42 mM CEES + 1.51 µM curcumin
6	10^4^ HCE-2 cells + 3.42 mM CEES + 20.22 µg/ml doxycycline
7	10^4^ HCE-2 cells + 3.42 mM CEES + 10.11 µg/ml doxycycline
8	10^4^ HCE-2 cells + 3.42 mM CEES + 5.05 µg/ml doxycycline
	
	
HCEC, human corneal epithelial cell, CEES, 2-chloroethyl ethyl sulfide	

**Table 2:** Doxycycline eye drop formulation.


**Substance**	**Role **	**Percentage **
Doxycycline hyclate	Effective material	2.3008 ${\;}^{*}$
HPMC	Covering polymer	0.15
NaCl	Isotonic factor	0.112
KCl	Isotonic factor	0.093
Sodium tetraborate	Buffer	0.048
Boric acid	Acidic agent	0.048
Water	Diluent	Up to 100 ml
	
	
HPMC, hydroxypropyl methylcellulose; NaCl, Sodium chloride; KCl, Potassium chloride ${\;}^{*}$ 1.154 gram of doxycycline hyclate is equivalent to 1 gram of doxycycline		

**Table 3:** Cell treatment method to evaluate the effect of curcumin and doxycycline on CEES cytotoxicity.


**Well no. (Group)**	**Treatment method and the contents of the wells**
1 (Control group)	10^5^ HCE-2 cells
2 (CEES group)	10^5^ HCE-2 cells + 3.42 mM CEES
3 (Curcumin group)	10^5^ HCE-2 cells + 3.42 mM CEES + 3 µM curcumin
4 (Doxycycline group)	10^5^ HCE-2 cells + 3.42 mM CEES + 10 µg/ml doxycycline
	
	
HCEC, Human corneal epithelial cell; CEES, 2-chloroethyl ethyl sulfide	

**Table 4:** The sequence of the primers used in qPCR.


**PCR product length**	**Primers Sequence**	**Primers**
200 bp	GCAACCTGTTTGTGCTGAAG	MMP-2 (Forward)
	GTAGCCAATGATCCTGTATGTG	MMP-2 (Reverse)
114 bp	TCCAGTACCGAGAGAAAGCCTA	MMP-9 (Forward)
	GCAGGATGTCATAGGTCACG	MMP-9 (Reverse)
174 bp	TGCGGATACTTCCACAGGTC	TIMP-1 (Forward)
	GCATTCCTCACAGCCAACAG	TIMP-1 (Reverse)
63 bp	TGTGCCGCTAGAGGTGAAATT	18s rRNA (Forward)
	TGGCAAATGCTTTCGCTTT	18s rRNA (Reverse)
	
	
MMP, matrix metalloproteinase; TIMP, tissue inhibitors of metalloproteinase		

**Figure 1:**
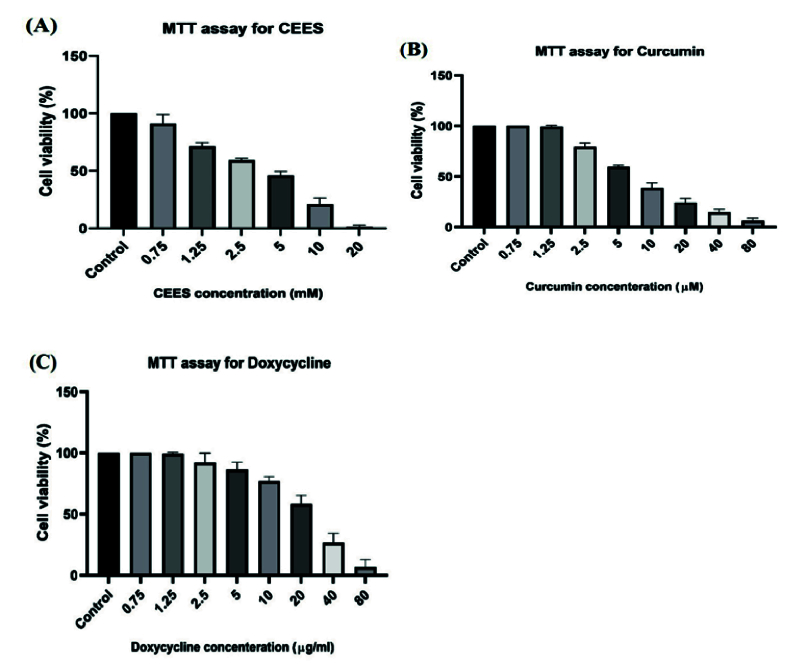
Cell viability in different concentrations of CEES (A), doxycycline (B), and curcumin (C).

**Figure 2:**
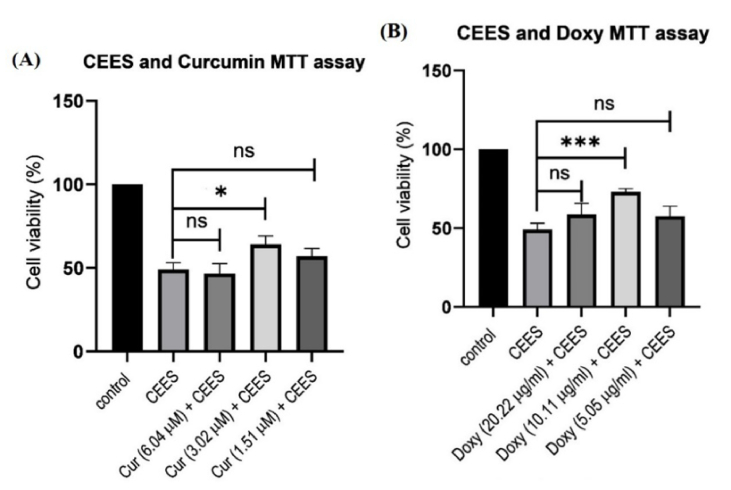
The impact of different and optimal concentrations of curcumin (A) and doxycycline (B) in inhibiting CEES.

**Figure 3:**
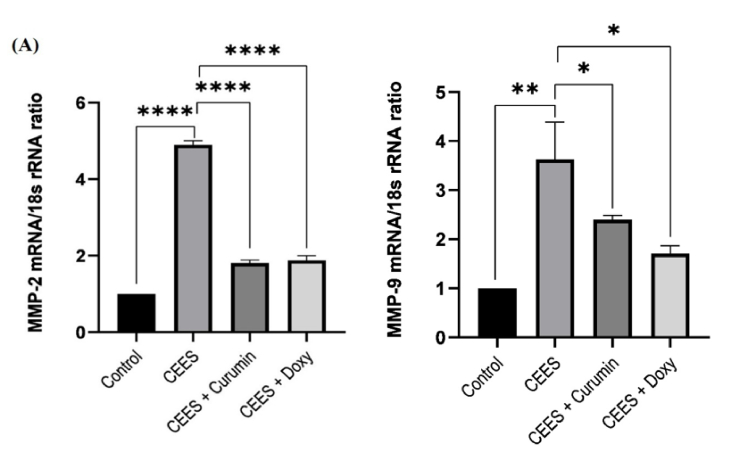
The expression levels of *MMP-2* (A) and *MMP-9 *(B) in the presence of CEES and after treatment with curcumin and doxycycline.

**Figure 4:**
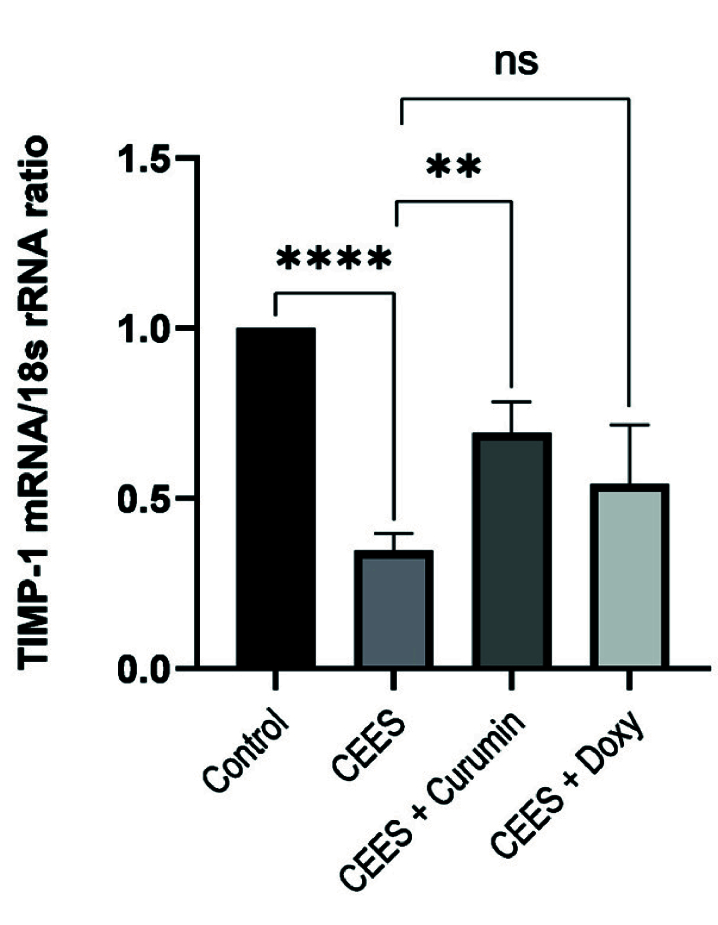
The expression levels of TIMP-1 in the presence of CEES and after treatment with curcumin and doxycycline.

**Figure 5:**
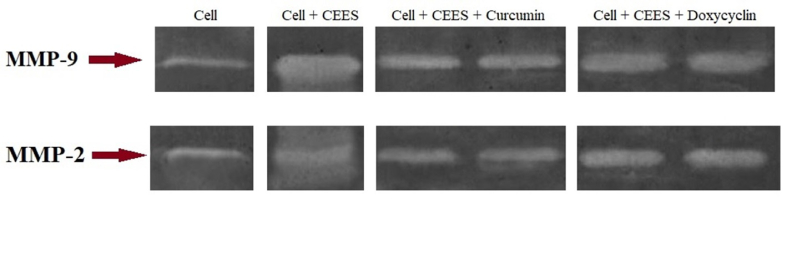
Activity of *MMP-2* and *MMP-9* in corneal epithelial cells in the presence of CEES and after treatment with curcumin and doxycycline, measured by zymography.

**Figure 6:**
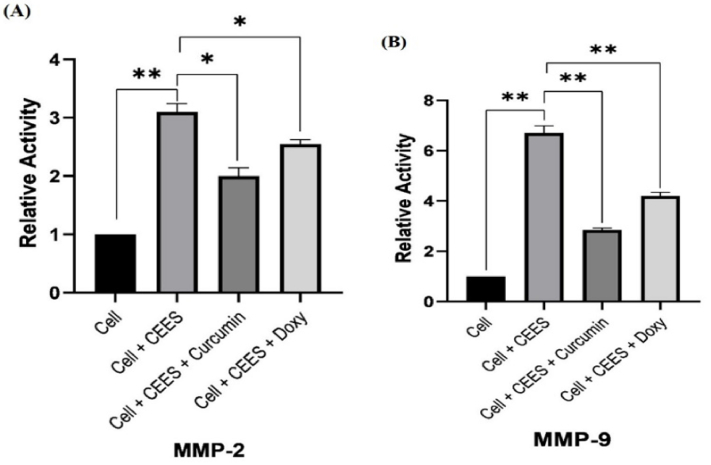
Relative activity of *MMP-2* (A) and *MMP-9* (B) in corneal epithelial cells in the presence of CEES and after treatment with curcumin and doxycycline, measured by zymography.

##  RESULTS

### IC50 Calculation

To determine the appropriate concentration of doxycycline and curcumin for treating the cells, we evaluated cell viability and the IC50 dose by the MTT assay. Figure 1 depicts cell viability in different concentrations of CEES, doxycycline, and curcumin. The calculated IC50 values for doxycycline, curcumin, and CEES were 40.44 µg/ml, 12.08 µM, and 3.42 mM, respectively. To evaluate the therapeutic effects of curcumin and doxycycline on CEES-infected corneal cells, we first exposed the cells to the IC50 concentration of CEES (3.42 mM) and then treated them with 10 µg/ml and 3 µM (one-fourth of IC50) of doxycycline and curcumin, respectively.

### Effective Doses of Doxycycline and Curcumin

To obtain the effective concentrations of curcumin and doxycycline for inhibiting CEES, we treated CEES-infected cells with different concentrations of these drugs. Next, the MTT test was performed to evaluate cell viability. (The results are shown in Figure 2.) The best concentrations of curcumin and doxycycline for CEES inhibition were 3.02 μ M and 10.11 μ g/ml, respectively.

### Evaluation of MMP-2, MMP-9, and TIMP-1 gene expression

When cells are exposed to CEES, the amount of MMPs increases in the cell, leading to tissue destruction. On the other hand, treating these cells with curcumin or doxycycline will reduce the toxic effects of CEES. Therefore, it is helpful to examine the amounts of these enzymes (*MMP-2* and *MMP-9*) and their inhibitor (TIMP-1). To this end, 24 hours after cell treatment, the expression levels of *MMP-2*, *MMP-9*, and TIMP-1 were assessed via qPCR. As seen in Figure 3A, the expression of *MMP-2* in corneal epithelial cells increased approximately 5-fold after their infection with CEES. Conversely, the expression of *MMP-2* declined 2.7-fold (*P* < 0.001) and 2.6-fold (*P* < 0.001) after treatment with curcumin and doxycycline, respectively. There was no significant difference between the effects of curcumin and doxycycline on reducing *MMP-2* expression.

According to Figure 3B, *MMP-9* showed a 3.3-fold higher expression in corneal epithelial cells after being infected with CEES, whereas the expression decreased 1.6-fold (*P* < 0.05) and 2.5-fold (*P* < 0.05) after treatment with curcumin and doxycycline, respectively. According to Figure 3B, the effect of doxycycline on reducing the activity of *MMP-9* is greater than that of curcumin, but the difference is not statistically significant.

Also, the expression of TIMP-1 mRNA in the CEES group significantly decreased compared with the control group [Figure 4]. After treatment with curcumin, the expression of TIMP-1 was 2-fold higher (*P* < 0.01) in corneal cells compared to the CEES group; however, no significant differences in the expression of TIMP-1 mRNA were observed between the doxycycline and CEES groups [Figure 4]. In other words, there was no significant difference between the effects of curcumin and doxycycline on increasing TIMP-1 expression.

### Evaluation of MMP Activity (Zymography)

The activity of *MMP-2* and *MMP-9* in corneal epithelial cells was determined by zymography. Dried gels were photographed, and the intensity and area of the band were measured using the Image Station 4000MM (Kodak Digital Science, United States). As shown in Figure 5, CEES heightened the activity of *MMP-2* and *MMP-9*. On the other hand, *MMP-2* and *MMP-9* activity decreased in the presence of 3.02 μ M of curcumin and 10.11 μ g/ml doxycycline. According to Figure 6, CEES increases the activity of *MMP-2* and *MMP-9* by about 3.1 and 6.9 fold, respectively. Treatment with curcumin reduced the activity of *MMP-2* and *MMP-9* by about 32% (*P* < 0.05) and 56% (*P* < 0.01), respectively, compared to the control group. Also, the activity of *MMP-2* and *MMP-9* decreased with doxycycline by about 12% (*P* < 0.05) and 40% (*P* < 0.01), respectively.

##  DISCUSSION

The eye, the most sensitive organ of the body, is often exposed to different injuries. SM causes a wide range of adverse effects when it comes into contact with the eye, highlighting the urgent need for treatment.^[21]^ CEES has a chemical structure similar to SM and it can similarly damage the eye. For this reason, it was considered in the present research.^[1]^

Cost-effectiveness and ethical issues are two factors that reduce the efficiency of animal models in the early stages of pharmaceutical research; therefore, we used a cell culture model for this study. In this investigation, an *in vitro* model was successfully developed for drug screening. The HCE-2 culture system allowed us to economically test several concentrations of curcumin and doxycycline on the infected corneal cell and, thus, assess their ability to inhibit the adverse effects of CEES. Immortalized HCE-2 from healthy cornea were infected by CEES and then treated with curcumin and doxycycline. To determine the best concentration of curcumin and doxycycline for CEES inhibition, we treated CEES-infected cells with three concentrations of these compounds and selected the optimal concentration according to the results [Figure 2].

SM-induced ocular damage involves processes such as inflammation, neovascularization of corneas, and ECM remodeling, in which the ECM is mainly regenerated by MMPs.^[22,23,24]^ Gelatinases (MMPs), mostly *MMP-2* and *MMP-9*, play a key role in the destruction of basal membrane components that impair epidermal connectivity in the eye and skin.^[1,25,26,27]^ Gordon et al showed that when ocular damage is caused by mustard toxin, *MMP-2* and *MMP-9* would play an important role in the separation of epithelial–stromal junctions in the ocular tissues.^[21]^ Indeed, during the development of acute injury, the activity of corneal *MMP-2* and *MMP-9* increases in tandem with the clinical manifestation of inflammation; but under normal circumstances, these MMPs are involved in the short-term, strongly regulated ECM remodeling.^[6,13,28]^ Also, TIMP-1 can inhibit the activity of MMPs.^[29]^ Therefore, we also examined the expression of this enzyme in this study.

According to the results of previous studies as well as the current study, it can be argued that inflammation-signaling pathways play a major role in SM-induced ocular damage.^[27]^ On the other hand, due to the involvement of *MMP-2* and *MMP-9* in the inflammatory process, we evaluated the impact of curcumin and doxycycline in treating ocular injuries.^[6]^ In this investigation, we evaluated the effect of curcumin and doxycycline on the expression (qPCR) and activity (zymography) of TIMP-1, *MMP-2*, and *MMP-9* in HCE-2 cells. According to the results, infection of cells with CEES heightens the expression and activity of *MMP-2* and *MMP-9*. The results of qPCR and zymography revealed that curcumin and doxycycline significantly reduced MMP expression and activity compared to the control group.

There was no significant difference between the effects of curcumin and doxycycline on reducing *MMP-2* expression, but the difference was significant in the case of *MMP-9*. According to the results, curcumin has a greater ability than doxycycline to inhibit *MMP-2* and *MMP-9* enzymes, yet the difference is statistically significant only in the case of *MMP-9*.

Marion et al tested four tetracyclines as a treatment against vesicants. Their results demonstrated that all tetracycline derivatives, including doxycycline, could inhibit *MMP-9* expression. Therefore, doxycycline may be effective against SM exposure due to its anti-MMP activity.^[21]^ In another study by Anumolu et al, an increase in *MMP-9* activity and severity of the injury was observed in rabbit corneas exposed to SM analogs. The authors reported that topical doxycycline treatment diminished *MMP-9* activity.^[6]^ Horowitz et al also evaluated the effect of doxycycline (2 mg/ml) on SM injury in rabbit eyes. They collected corneal and tear samples at different times and measured MMP activity by zymography. The authors found that long-term treatment with doxycycline reduces the activity of *MMP-9* in the tear fluid, leading to decreased severity of corneal damage during the acute phase.^[6]^

Banerji et al reported that 15 mM curcumin has a significant inhibitory effect on *MMP-2* activity in metastatic murine melanoma cells B16F10. Interestingly, this inhibitory effect persisted for 28 days after curcumin removal.^[30]^ In another study, Shao et al reported that curcumin has a strong anti-invasive effect on human breast carcinoma cells. Their result showed that this anti-invasive effect is related to downregulation of *MMP-2* and upregulation of TIMP-1.^[31]^ Also, Lin et al showed that curcumin reduced the levels of *MMP-2* and *MMP-9* in adenocarcinoma human epithelial cells (A549 cell line).

Chao et al explored the effects of curcumin on the secretion of interleukin 6 (IL-6) and interleukin 8 (IL-8) by human corneal limbal epithelial cells. The cells were irradiated by UVB at different dosages with or without curcumin. Next, the levels of IL-6 and IL-8 in the cells were measured by ELISA analysis. According to the results, curcumin (5-20 µmol/L) significantly inhibited UVB-induced secretion of IL-6 and IL-8 by limbal epithelial cells in a dose-dependent manner. Consequently, the authors suggested, curcumin may be a promising agent to be explored for the prevention and treatment of pterygium.^[32]^

In another study, Chen et al confirmed the anti-inflammatory effects of curcumin in dry eye disease. Specifically, the increase in osmolarity caused by the addition of sodium chloride to the culture medium of corneal epithelial cells enhances the production of IL-1 β (a pro-inflammatory cytokine). The authors investigated the anti-inflammatory effects of curcumin in this cell model, and they observed that curcumin pretreatment (5 μ M) eliminated the increase in IL-1 β production induced by the hyperosmotic medium. Accordingly, it appears that curcumin may have a therapeutic potential for dry eye disease.^[33]^

One of the most important factors that contribute to corneal hemostasis is the barrier function of the corneal epithelium, which is disturbed by inflammation. Whereas tight junctions and adherents junctions of the corneal epithelium are required for the barrier function and cell adhesion, some inflammatory cytokines such as IL-1 β and TNF can disrupt the function of this barrier. Kimura et al, in two separate studies, investigated the therapeutic effect of curcumin on these two cytokines. They found that curcumin blocked the effects of IL-1 β and TNF- α on both TER and the subcellular localization of ZO-1. Finally, the authors proposed, curcumin can be used as an anti-inflammatory agent for eye diseases.^[34,35]^

In summary, the results of the present study showed that curcumin could reduce the expression and activity of *MMP-2* and *MMP-9* after their induction by CEES. The observed effectiveness of curcumin in inhibiting MMPs compared to doxycycline confirms its positive role as a natural substance in treating CEES-induced ocular inflammation. Further investigation is needed to confirm the safety and anti-inflammatory activity of curcumin under *in vivo* conditions.

### Financial Support and Sponsorship 

This work was supported by the Baqiyatallah University of Medical Sciences (Grant number: 97000503).

##  Conflicts of Interest

None.
